# Supplementary material for: Which mouse multiparental population is right for your study? The Collaborative Cross inbred strains, their F1 hybrids, or the Diversity Outbred population
Source: G3 (Bethesda). 2023 Feb 3;13(4):jkad027. doi: 10.1093/g3journal/jkad027 (PMC10085760; doi:10.1093/g3journal/jkad027)
Supplement: jkad027_Supplementary_Data [file jkad027_supplementary_data.pdf]

Supplemental material for

"Which mouse multiparental population is right for your study? The Collaborative Cross inbred strains, their F1 hybrids, or the Diversity Outbred population"

Gregory R. Keele

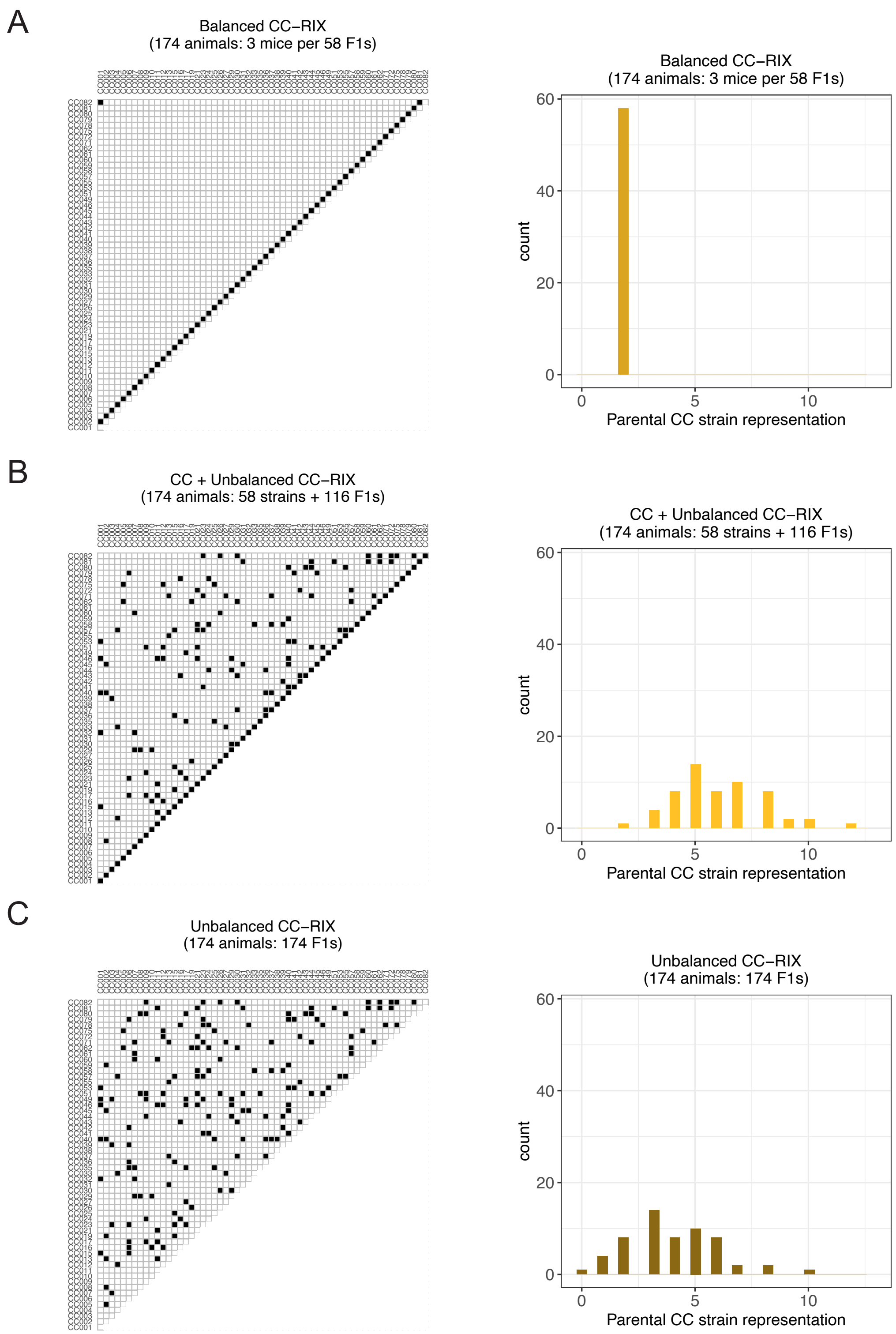

Figure S1 Examples of CC-RIX sample populations (as used in Figure 3C-E): (A) a balanced F1 set where each CC parental strain is equally represented twice, (B) an unbalanced F1 set combined with the CC strains, and (C) an unbalanced set of CC-RIX F1s alone. Each population is represented with a diallel grid of the CC strains and their F1s (left column) and a histogram of the how many F1s/strains represent each parental CC strain (right column). Selected strains and F1s are denoted as black cells in the diallel grid. The effects of sex chromosomes and parent-of-origin are ignored.

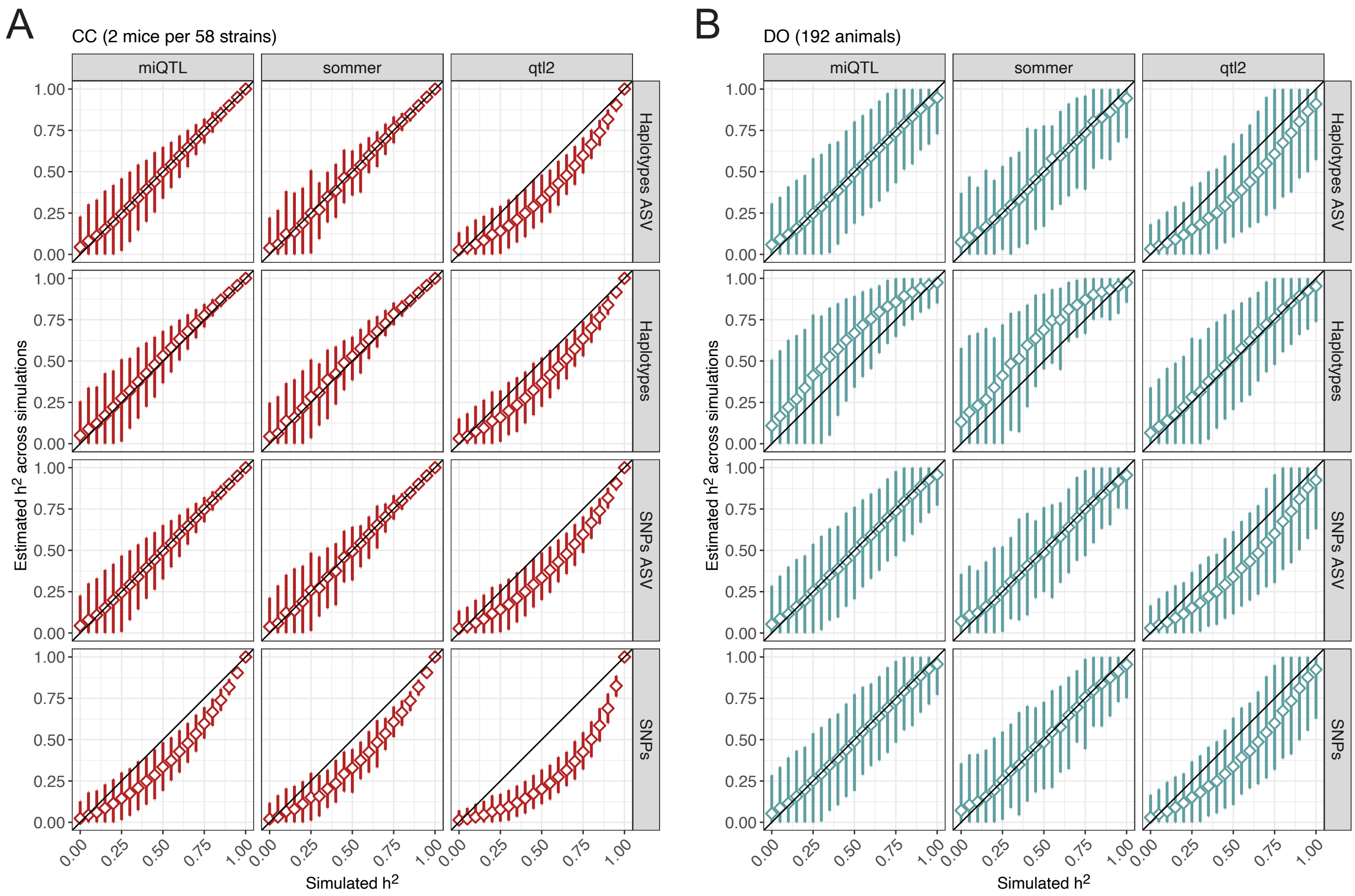

Figure S2 Performance of heritability estimation across kinship matrix estimates (rows) and software packages (columns) in data simulated for (A) 116 CC mice (two per strain) and (B) 192 DO mice. Diamonds represent the mean estimated heritability across simulations from the true heritability. 1,000 simulations were performed with qtl2 and miQTL and 100 were performed for sommer. Vertical line segments represent middle 95% intervals across the simulations. Black diagonal lines indicating the heritability estimate is equal to the true value (y = x) included for reference.

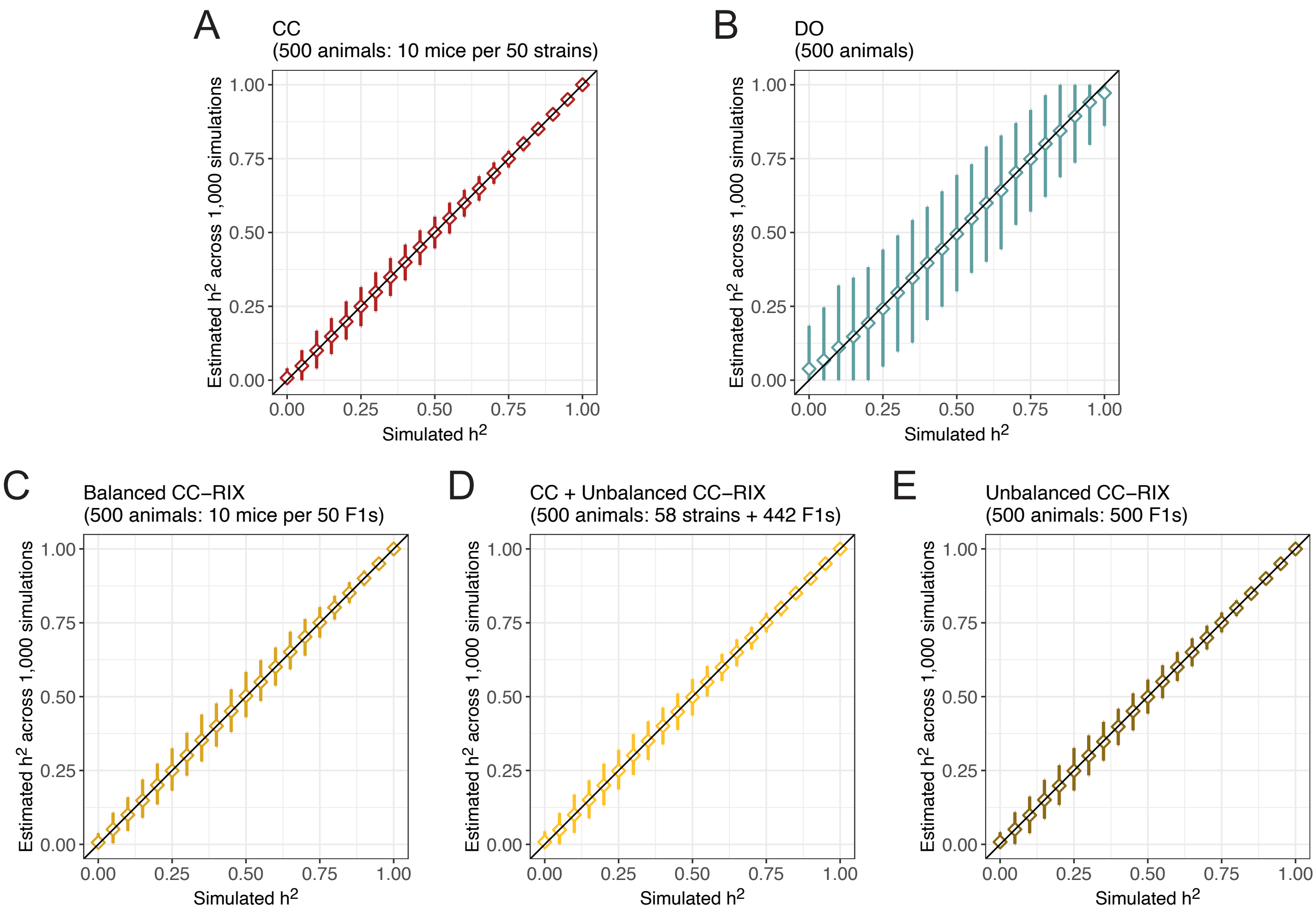

Figure S3 Performance of heritability estimation in data simulated for 500 mice from (A) CC, (B) DO, and (C-E) CC-RIX populations. Diamonds represent the mean estimated heritability across 1,000 simulations from the true heritability. Vertical line segments represent middle 95% intervals across the 1,000 simulations. Black diagonal lines indicating the heritability estimate is equal to the true value (y = x) included for reference. See Figure 3 for results from simulations of 174 mice.

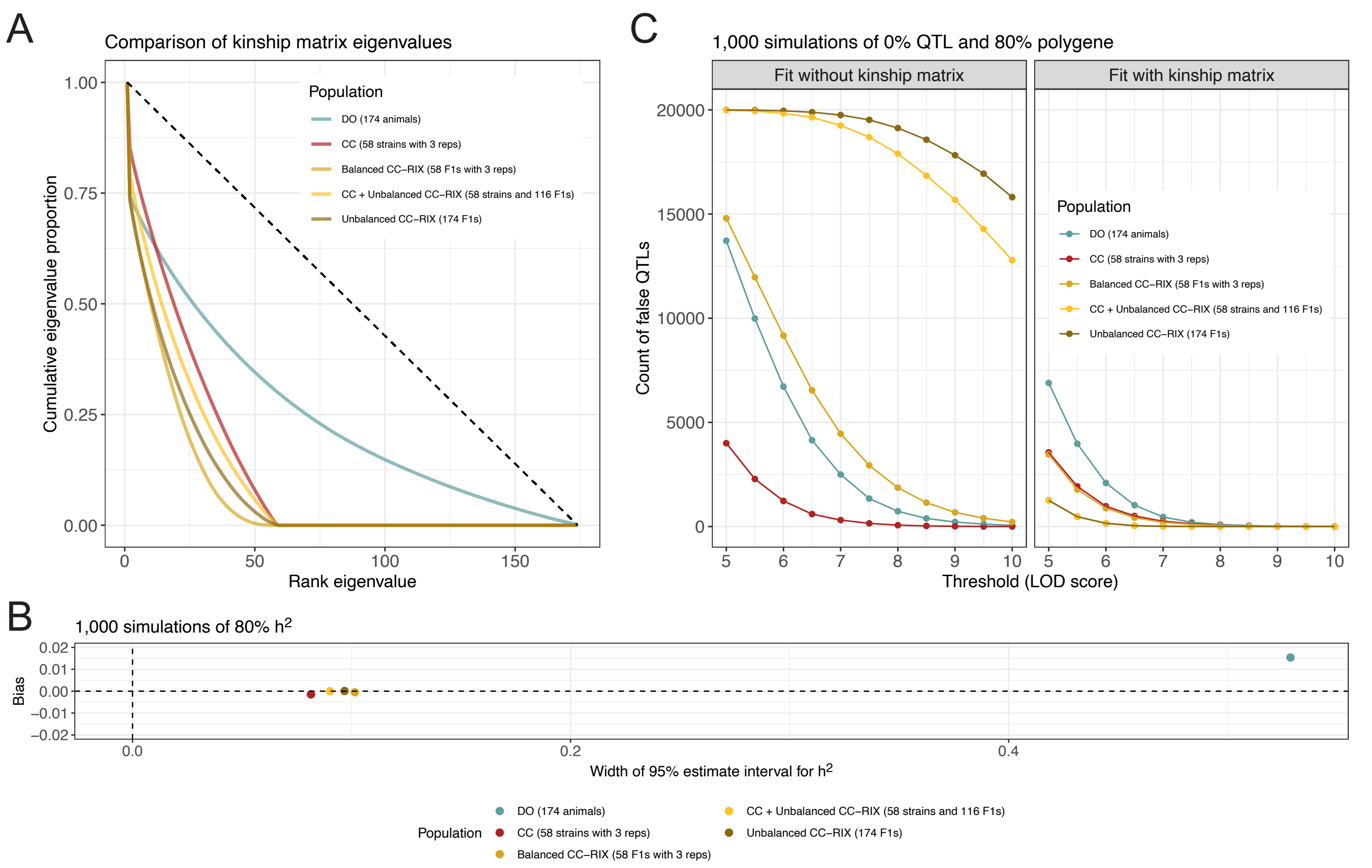

Figure S4 Population structure or unequal relatedness influences heritability estimation and QTL mapping. (A) Population structure summarized as cumulative eigenvalues (y-axis) by the rank of the eigenvalues of the kinship matrices for the CC, CC-RIX, and DO populations. Diagonal dashed line included for reference representing a population with no structure. (B) Comparison of heritability estimation bias (y-axis) to its precision in the form of 95% estimate interval width (x-axis) across the populations. Summaries for each population are based on 1,000 simulations with heritability set to 80%. Horizontal and vertical dashed lines at 0 included for reference representing a perfect summary with no bias or uncertainty. (C) Population structure can result in false positive QTLs when it is not accounted for in the model. The number of false QTLs were counted from 1,000 simulations of data with no QTL and 80% polygenic effect size for all populations.

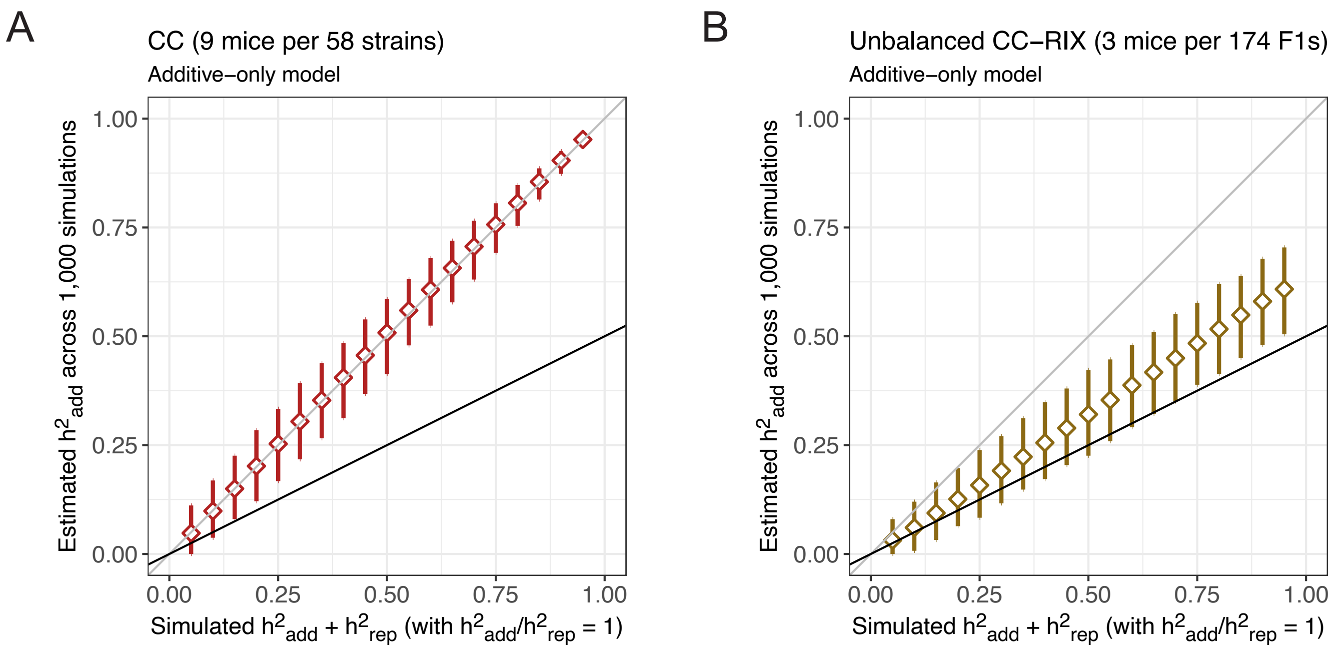

Figure S5 Additive and strain-specific genetic effects are confounded in the CC, but less so in the CC-RIX. Performance of heritability estimation in data simulated for 522 mice from (A) CC and (B) CC-RIX populations with additive (h^2_{add}) and strain/F1 (h^2_{rep}) components but the fit model is miss-specified with only an additive component. Diamonds represent the mean estimated heritability across 1,000 simulations from the true heritability. Vertical line segments represent middle 95% intervals across the 1,000 simulations. Black lines indicating the additive heritability component estimate is equal to the true value (y = 2x) included for reference. Gray lines indicating the additive heritability component estimate is equal to the true value of the sum of heritability components (y = x) included for reference.

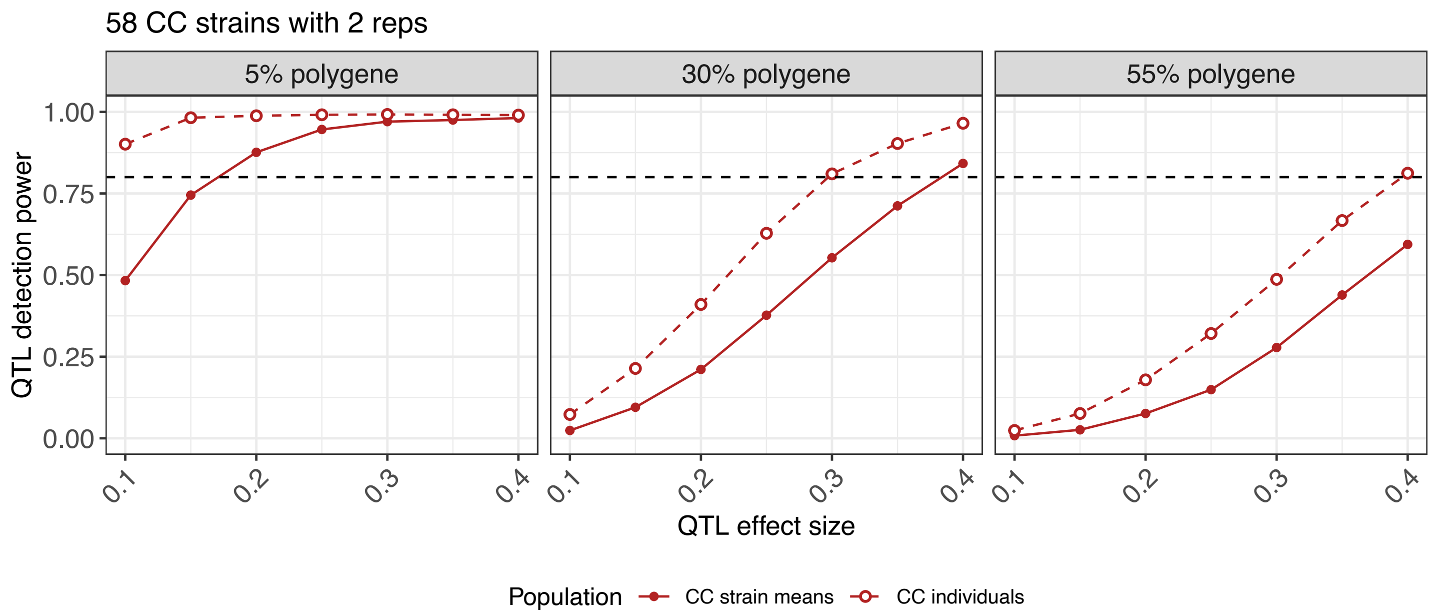

Figure S6 Comparison of QTL mapping power between mapping on CC strain means or the individual-level data. Power was summarized over 1,000 simulated QTLs using genome-wide significance thresholds across low-to-moderate polygenic backgrounds (columns). Simulations were of 116 animals (two animals per 58 CC strains). Horizontal dashed lines at 80% power included for reference.

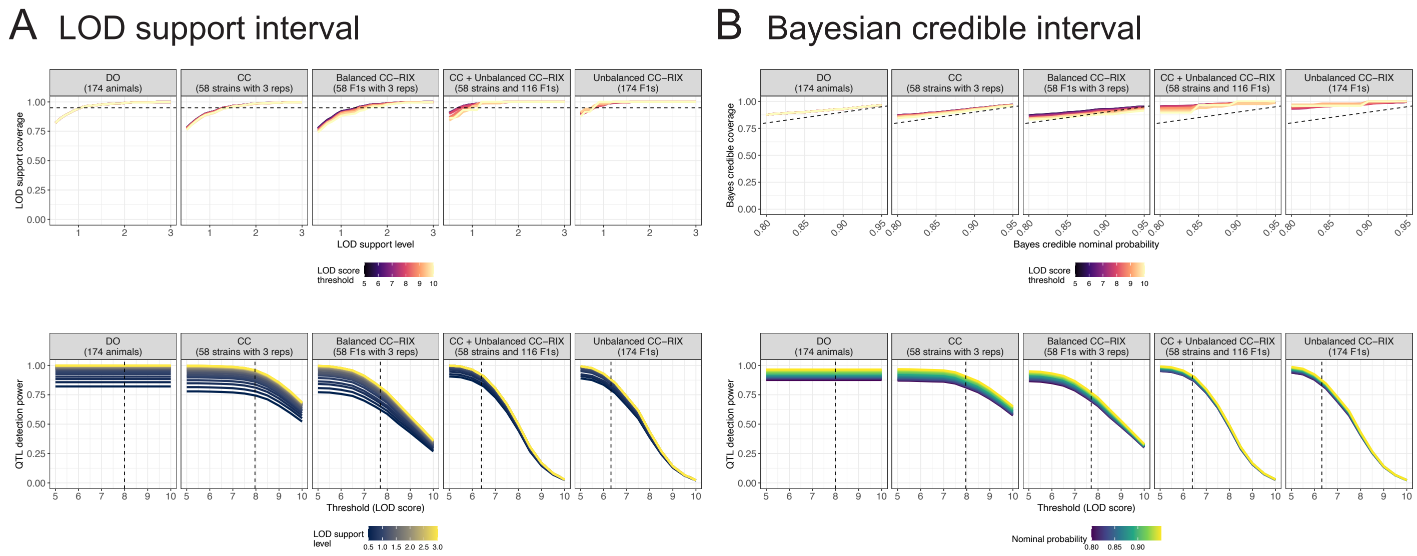

Figure S7 Performance of likelihood-based intervals for QTL location for CC, CC-RIX, and DO populations in terms of (top) QTL coverage rate and (bottom) mapping power. Likelihood-based approaches included (A) LOD support intervals and (B) Bayes credible intervals. Intervals are summarized over 1,000 QTLs (40% QTL with 30% polygenic background) simulated in 174 animals for each sample population. For the coverage rate of LOD support intervals, horizontal dashed lines at 80% coverage included for reference. For the coverage rate of Bayes credible intervals, diagonal dashed lines indicating the interval coverage rate is equal to the nominal probability (y = x) included for reference. For mapping power, vertical dashed lines represent the genome-wide 95% significance thresholds for each population. See Figure S8 for results from sampling-based intervals.

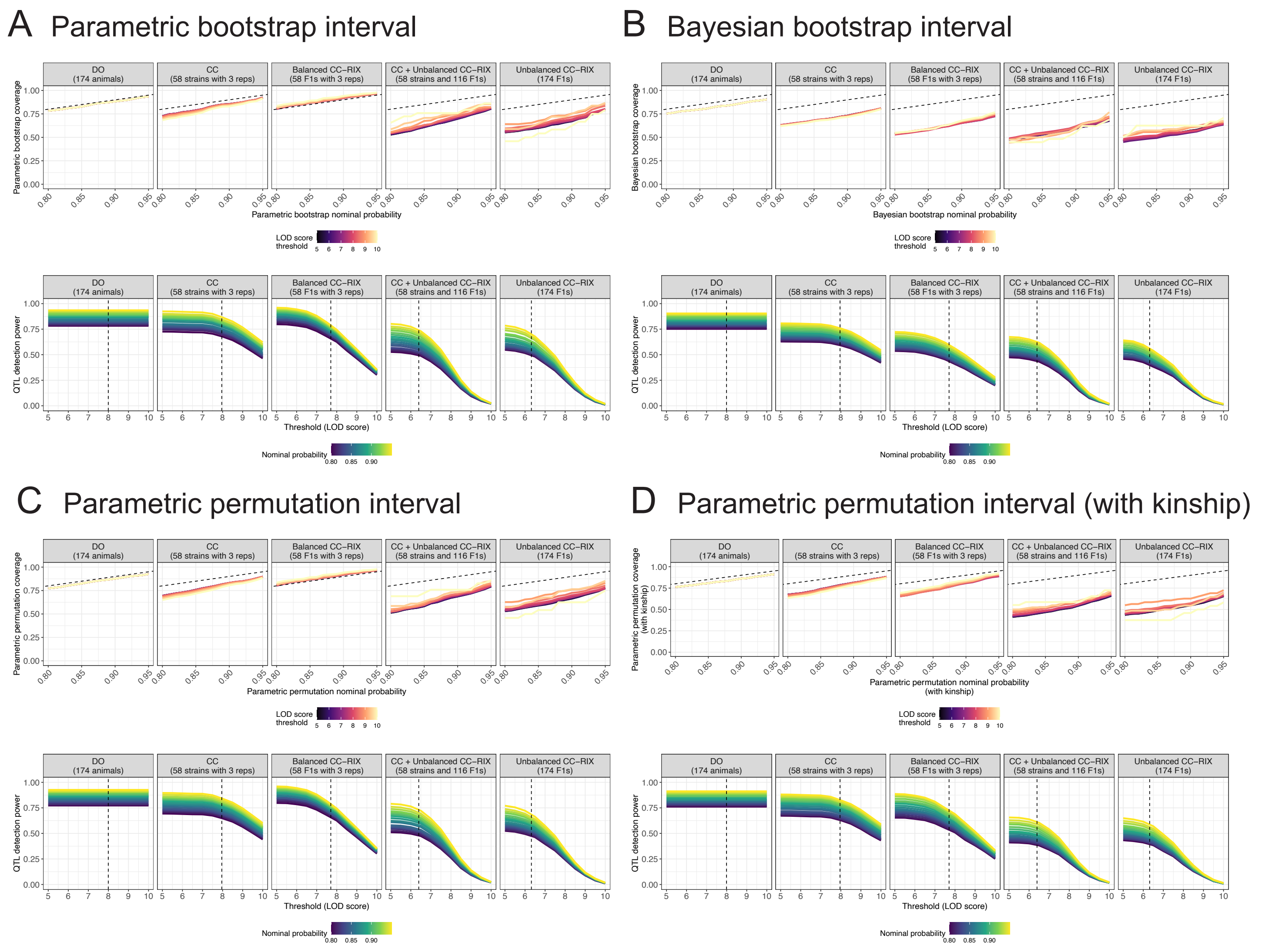

Figure S8 Performance of sampling-based intervals for QTL location for CC, CC-RIX, and DO populations in terms of (top) QTL coverage rate and (bottom) mapping power. Sampling-based approaches included (A) parametric bootstrap intervals, (B) Bayesian bootstrap intervals, (C) parametric permutation intervals, and (D) parametric permutation intervals with kinship included. Intervals are summarized over 1,000 QTLs (40% QTL with 30% polygenic background) simulated in 174 animals for each sample population. For Bayesian bootstrap and parametric permutation intervals with kinship, 200 samples were generated; for the other interval types, 1,000 samples were used. For coverage rate, diagonal dashed lines indicating the interval coverage rate is equal to the nominal probability (y = x) included for reference. For mapping power, vertical dashed lines represent the 95% significance thresholds for each population. See Figure S7 for results from likelihood-based intervals.

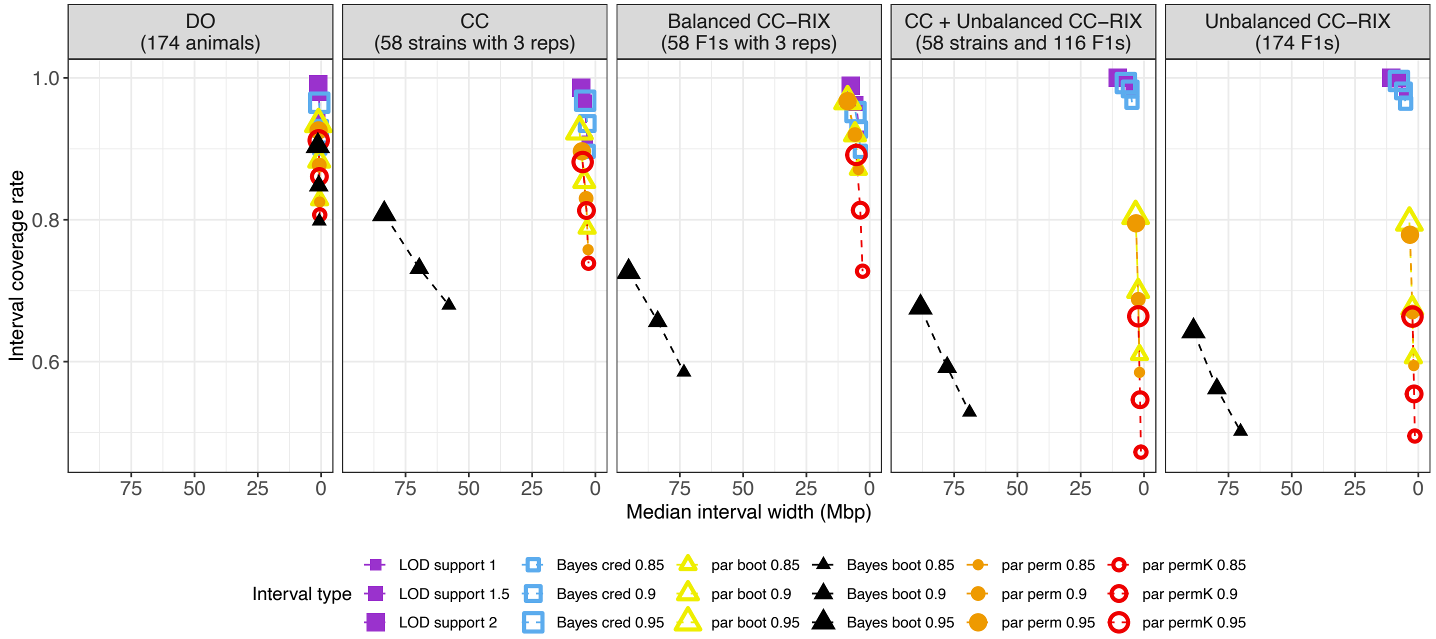

Figure S9 Performance of QTL location intervals across data simulated for CC, CC-RIX, and DO populations in terms of QTL coverage rate (y-axis) and interval width (x-axis). Intervals are summarized over 1,000 QTLs simulated in 174 animals for each sample population (using the 40% QTL and 30% polygenic background setting). Cool colors represent likelihood-based intervals: LOD support (purple) and Bayes credible (Bayes cred; blue). Warm colors represent sampling-based intervals: parametric bootstrap (par boot; yellow), parametric permutation (par perm; orange), and parametric permutation with kinship matrix (par permK; red). Another sampling-based interval, Bayesian bootstrap (Bayes boot) is colored black. Dashed lines connect summaries from the same procedure but with differing support levels, with increasing support indicated by larger symbols. For Bayesian bootstrap intervals and parametric permutation intervals with kinship, 200 samples were generated; for the other sampling-based intervals, 1,000 samples were used. The same summaries are shown in Figure 9 with Bayesian bootstrap intervals omitted to increase clarity.
